# HIV Voluntary Counselling and Testing Utilisation among School of Healthcare Sciences Undergraduate Students at the University of Limpopo

**DOI:** 10.3390/ijerph21020183

**Published:** 2024-02-06

**Authors:** Melitah Molatelo Rasweswe, Mamare Adelaide Bopape, Tshepo Albert Ntho

**Affiliations:** Department of Nursing Science, University of Limpopo, Private Bag X 1106, Sovenga, Polokane 0727, South Africa; mamare.bopape@ul.ac.za (M.A.B.); tshepo.ntho@ul.ac.za (T.A.N.)

**Keywords:** healthcare, HIV, Limpopo, undergraduate students, university, voluntary counselling and testing

## Abstract

Existing evidence indicates that South African university students have low utilisation of Human Immunodeficiency Virus (HIV) Voluntary Counselling and Testing (VCT). A cross-sectional survey was conducted to determine the utilisation of HIV VCT among undergraduate students in the School of Healthcare Sciences. Structured questionnaires were used to collect data through Google Forms. The results are presented through descriptions and percentages and illustrated in tables. Out of 389 undergraduate students, only 324 completed the questionnaire. The majority (97.2%) were aware of the health centre on campus, while only (74.7%) knew about the HIV VCT services offered on campus. Despite the awareness, many (36.7%) do not utilise the campus HIV VCT services, and some (9.6%) have never tested for HIV. There was an association between awareness of Voluntary Counselling and Testing of HIV services offered at the campus health and wellness centre and utilisation of HIV Voluntary Counselling and Testing at (<0.001). Therefore, it is imperative to urgently escalate the level of HIV/AIDS education in higher institutions of learning and emphasise the mounting danger of HIV infection and the immense importance of regular HIV testing. The findings of this study could serve as a foundation for creating HIV prevention and control programmes for youth, particularly in higher education institutions.

## 1. Introduction

The spread of Human Immunodeficiency Virus (HIV) and Acquired Immunodeficiency Syndrome (AIDS) among young people is a significant global health concern [1]. Thus, knowing HIV status is crucial in preventing the spread of the virus and promoting early treatment and care. In South Africa, Voluntary Counselling and Testing (VCT) has been a highly effective intervention towards achieving these goals [2]. People who receive VCT services are informed about their status and how to protect others and themselves by abstaining from dangerous sexual behaviours. Additionally, VCT provides access to HIV care and treatment, as well as emotional support that helps people manage their anxiety related to HIV and make plans for the future. There are also several measures to increase the utilisation of VCT for free among young people, including Khomanani, Love Life, Soul Buddz, Soul City television show series, and other promotions [3]. There are also youth-friendly HIV testing services available in most healthcare facilities. In addition, Higher Education and Training HIV/AIDS [HEAIDS] was established to encourage students in Higher Education Institutions to easily access and utilise the HIV services within their learning premises. By the end of 2015, 74% of advanced education organisations in SA had laid out HIV testing administrations, with 69% of these establishments offering the services for free [4]. The University of Limpopo provides free VCT as part of the government’s HEAIDS programme.

Many studies conducted globally among university students on HIV VCT show that students have not been aware of and utilised many of these services. For example, in Thailand, the acceptance of VCT among Thai university students was lower [5]. A similar problem was experienced in sub-Saharan African universities. In a Nigerian university, it was found that although students know about VCT, they do not use it efficiently, as only a few students reported having tested for HIV at the university VCT services [6]. The study conducted among Bahirdar University students in Ethiopia has shown a significant lack of awareness and low uptake of Voluntary Counseling and Testing (VCT) services. This highlights the need for increased education and awareness campaigns to increase the uptake of VCT services among the student population. [7]. In a public University in the Volta Region of Ghana, about a third of first-year students were unwilling to test for HIV [8]. Also, the literature examination revealed that the rate of VCT is low in South African universities. For instance, in 2012, the University of Kwa-Zulu Natal highlighted students’ low utilisation of VCT [9]. The University of Limpopo reported similar results in 2014 [10]. The most current study conducted at the University of Venda showed that despite high knowledge of HIV testing services (HTS) by students between the ages of 18 and 24, only (44%) were tested for HIV as compared to (56%) who were not tested [11]. Both studies link the low utilisation to other various factors. Studies have found that there are various reasons why the uptake of VCT services is low. These include the stigma and breach of confidentiality that are linked with these services, as well as the fear of blood, pain, and needles when undergoing testing [5]. Additionally, the fear of knowing one’s own HIV status and a lower HIV transmission knowledge score have also been cited as reasons for the low uptake [5,11,12]. According to Sambou and Colleagues, the available evidence on the factors influencing or impacting HIV VCT uptake remains insufficient and conflicting [13]. This is of concern at the University of Limpopo because the primary cause of HIV infection in South Africa is sexual contact, and an increasing proportion of people living with HIV in the country have been reported to continue having unprotected sex [14,15]. Young people between the ages of 15 and 24 years, including university students in South Africa, are at higher risk of new HIV infections because they also engage in risky sexual behaviours such as unprotected sex with people who are older than them by five or more years [16,17].

In South Africa, it is estimated that there are currently 7.8 million people living with HIV [18]. The prevalence of HIV among younger populations shows significant differences in terms of gender. For instance, females aged between 15 and 19 years have approximately twice the HIV prevalence rate compared to males of the same age group (5.6% vs. 3%, respectively) [18]. According to recent statistics by the South African Human Sciences Research Council, the prevalence of HIV among people aged between 15 and 49 years in Limpopo Province is approximately 11.9% [18]. Limpopo is among the poorest-performing provinces regarding the 95-95-95 targets [19]. All these put university students at higher risk of being infected with HIV. Hence, this study aimed to determine HIV VCT and associated factors among undergraduate students from the School of Healthcare Science at the University of Limpopo.

## 2. Materials and Methods

### 2.1. Study Design and Setting

This study followed a cross-sectional survey design that was implemented to determine the HIV VCT utilisation and associated factors among School of Healthcare Science undergraduate students at the University of Limpopo, Turfloop campus, in Mankweng Township, South Africa. The university is situated in the Capricorn district of Limpopo Province, along R71 Tzaneen Road. It is about 30 kilometres from the eastern side of Polokwane, the capital city of Limpopo Province. The University of Limpopo enrols students from all nine provinces of South Africa and neighbouring countries, such as Zimbabwe and Mozambique. This university is one of the previously disadvantaged universities, and most students are from disadvantaged families. The university has four faculties, including Health Sciences. The faculty of Health Sciences consists of 2 schools: the School of Medicine and the School of Healthcare Sciences. The School of Healthcare Sciences consists of 5 departments: Nursing, Optometry, Human Nutrition and Dietetics, Pharmacy, and Public Health. A health and wellness centre on the campus provides health services, including VCT, a support system for people living with HIV, and other services to the university community.

### 2.2. Study Population

The target population was all the undergraduate students enrolled in the School of Healthcare Sciences, excluding public health, for the 2023 academic year. A simple random probability sampling technique was used to ensure that all the School of Healthcare Sciences undergraduate students had an equal chance of being included. To ensure the representativeness of the data proportional to the number of students, we looked into the total number of students enrolled per department and allocated a percentage to be sampled. A sample of 389 was obtained using Slovin’s formula (1960), based on desired accuracy with a confidence level of 95 percent and a margin error of 0.05. However, only 324 responded.

### 2.3. Procedure

Data were collected between August and October 2023. We created an online questionnaire using Google Forms to collect the data. Students were recruited through their university email addresses. An overview of the study objectives, researcher contact details, an online consent form, and a link to access a questionnaire were shared in the recruitment email. The students were required to click a button at the end of an online consent form to participate and gain access to the questions.

### 2.4. Data Collection Instrument

The research instrument comprised a self-administered questionnaire constructed in English by the authors, guided by the relevant literature and Andersen’s Behavioral Model (ABM) of Health Service Use to guide the selection of independent variables. Independent variables included all demographic characteristics. All the questions were closed-ended. The questions in the survey were designed in such a way that the students had to respond with either “Yes” or “No” to some questions, while others required them to indicate if they “Agree”, “Disagree”, or “Do not know”. Some questions were presented as multiple-choice, allowing the students to select the most relevant option. The survey comprised two sections: (1) Sociodemographic, which included gender, age, level of study, and department of enrollment; and (2) Utilisation of HIV Voluntary Counselling and Testing which was measured using a 7-item questionnaire. The questionnaire assessed the respondent’s awareness of the campus health and wellness centre, their knowledge of VCT services offered at the centre, their willingness to use the HIV VCT services at the campus health and wellness centre, their frequency of VCT utilisation, their experience with voluntary HIV testing, and their reasons for getting tested.

### 2.5. Data Analysis

The Google Form system generated, cleaned, and analysed datacompletion, which was then extracted to an Excel spreadsheet. During the analysis, both descriptive and inferential statistics were used. A Pearson Chi-square test was used to ascertain the relationship between the variables, including respondents’ demographic characteristics and HIV VCT utilisation. This study’s threshold of significance was set at a *p*-value of 0.05. Numerical data were combined, interpreted, and communicated using descriptive statistics summarised in frequencies and percentages.

### 2.6. Ethical Issues

Ethical approval to conduct this study was issued by the Turfloop Research Ethics Committee (TREC No. 525/2023: UG). Permission was obtained from the school director and each Head of Department in the School of Healthcare Sciences at the University of Limpopo. All the respondents provided informed consent to participate before completing a questionnaire.

## 3. Results

### 3.1. Demographic Characteristics

Table 1 presents the demographic information. The data indicates that most respondents were women, accounting for 71% of the total population. Men, on the other hand, constituted 25.9% of the participants. The remaining 3.1% opted not to reveal their gender. Out of 324 respondents, the most (52.8%) were between the ages of 18 and 21, followed by (46%) who were between 22 and 25 years old, and the least (1.2%) of respondents were 17 years and younger. The highest number of undergraduate students (38.3%) were at the fourth level, followed by the third level n = 98 (30.2%), level two (16.6%), and level one the least (15.1%). The majority of the undergraduates who responded were from the nursing department (40.7%), followed by the pharmacy students (25.9%), while the least (17.8%) were from the department of optometry and (15.7%) were from the department of dietetics.

### 3.2. Utilisation of HIV Voluntary Counselling and Testing

The utilisation of HIV voluntary counselling and testing among the undergraduate students in the School of Healthcare Sciences was assessed through awareness of the campus health and well-being centre, services offered, frequencies to utilise and declaring intentions to utilise the services at the campus health and wellness centre in the future. It also checked if the students utilise HIV VCT outside the campus, if they have ever voluntarily tested for HIV before, and the reasons for testing. Table 2 present the respondents views on utilization of HIV Volutary Counselling and Testing. Out of the n = 324 (100%) respondents, the majority (97.2%) were aware of the health centre on the campus. However, there were still (2.8%) who were not aware. Most respondents (74.7%) indicated they know the HIV VCT services offered within the campus health and wellness centre. What is disturbing is that there are still (25.3%) of students who are not aware of the HIV VCT services offered. Of concern is that (36.7%) do not utilise the campus HIV VCT services, while (7.1%) of respondents are utilising them more than three times in 12 months. The majority (60.2%) are willing to utilise on campus HIV services in the future, while a high percentage (30.9%) do not know if they will use them.

Regarding utilisation of the HIV VCT outside the campus, (12%) did not respond to the question, while many of the respondents (44.8%) are not utilising it and (43.2%) are utilising the outside campus services. Out of 324 respondents, only n = 293 (90.4%) had voluntarily tested for HIV before, meaning that (9.6%) never attempted to know their HIV status. The majority of respondents (64.2%) indicated the reason for testing is to know their own HIV status, followed by (10.2%) who think you only get tested if engaged in unprotected sex. In comparison, a few n = 25 (7.7%) are saying testing is for sick individuals, some (9.6%) chose to go for testing if getting into a new relationship, and (8.3%) chose only if advised by a health care provider.

### 3.3. Association between Variables and Utilisation of HIV Voluntary Counselling and Testing

The results, as illustrated in Table 3, show that there is no association between demographic characteristics and the utilisation of HIV Voluntary Counselling and Testing. A statistically significant association was found between awareness of the Voluntary Counselling and Testing of HIV services offered at the campus health and wellness centre and utilisation of HIV Voluntary Counselling and Testing (Chi-Square = 42.620; *p*-value ≤ 0.001). This study’s threshold of significance was set at a *p*-value of 0.05.

## 4. Discussion

This paper reported utilising HIV Voluntary Counselling and Testing among School of Healthcare Sciences undergraduate students at the University of Limpopo. The utilisation of HIV Voluntary Counselling and Testing services is an indicator for prevention, promotion of early treatment, and more prolonged survival. Unlike the study that scored only (45.5%) on awareness of the HIV prevention services offered on campus [20], the current results show that the majority of those surveyed (97.2%) were aware that there was a health centre on campus and (74.7%) knew that HIV VCT services are provided by it. The results are nearly similar to the study that scored (84.4%) on the awareness of health centres on campus and that HIV services were available [21]. Literature reported that many other tertiary students scored highly on awareness of HIV services offered on campus [11]. The high level of awareness could be attributed to the sustained and improved health education programmes offered by the campus health and wellness centre staff at the University of Limpopo. Over a few years, the University of Limpopo campus health and wellness centre and its partners raised HIV VCT awareness among university students through various initiatives, such as the First Things First campaigns presented each semester. This is in line with the previous suggestion that more students utilise HIV services through outreach programmes [22]. Awareness of the available HIV services and where to get tested boosts one’s likelihood of taking an HIV test, although a disturbing percentage (30.9%) do not know if they will use the on-campus HIV services in the future. Therefore, to effectively encourage more tertiary students to be educated about HIV and utilise services to prevent sexually related infections, such initiatives should be reinforced by the introduction of more HIV interventions in tertiary institutions and providing those who undergo testing with incentives. Government policies that offer incentives for HIV testing in sub-Saharan Africa have an impact on the high uptake of VCT [13]. Hence, (90.4%) of our respondents reported having tested for HIV previously. Comparable to the research carried out at a public institution in Kenya, most students (86.2%) have undergone HIV testing [21]. The high percentage of students who tested for HIV in our study might have been influenced by the fact that they are healthcare sciences students and understand the importance of VCT. Surprisingly, in Kenyan public universities, students from the non-health science department were 1.38 times more likely to take the test than those from the health science department [21]. Our results are also unlike those of Italian undergraduate university students, where the majority of them (83.8%) never undergone HIV testing during their lifetime [23].

The results on the reasons for HIV testing suggest differences in one way or another. Knowledge of one’s status was a common reason for testing among undergraduate students from the University of Limpopo, School of Healthcare Sciences (64.2%). This percentage implies that students have other motivations behind HIV testing. This outcome contrasts with the findings, which showed that only (29.3%) of students who had tested for HIV needed to know their status [24]. However, the percentage is lower than the (79%) reported amongst health sciences students from the University of Venda [11]. Knowing one’s own HIV status is very crucial since, in the event of accidents, it will safeguard one’s intimate partners and others in their vicinity. While many students reported having tested for HIV, the testing frequency is concerning as it varies from 1 to 12 months, with (36.7%) of students not falling into any of the specified ranges. Students at the University of KwaZulu-Natal Howard College reported having tested for HIV at least once in the previous 12 months, which is consistent with our findings [25].

Regarding the uptake of outside-the-campus HIV VCT services among university students, our study reported that more than half (50.9%) are not utilising them. These results are almost similar to the study conducted at Mizan-Tepi University in southwestern Ethiopia, with 42.5% of those who are not utilising the off-campus HIV VCT Services [25]. The study conducted by Mwangi, Ngure, Thiga, and Ngure also reported a high number of university students not utilising the HIV VCT outside the campus [24]. Additionally, the students who were aware of the Voluntary Counselling and Testing of HIV services offered at the campus health and wellness centre (74.7%) were strongly associated with the utilisation of HIV Voluntary Counselling and Testing compared to their counterparts.

Nonetheless, some authors argue that there is a notable discrepancy in the use of HIV services among distinct demographics such as gender, age, socio-economic status, educational level, race, and others [26,27,28]. The current results revealed that gender, age, health department, and level of study were not associated with the utilisation of HIV VCT. In some comparative studies, while the level of study was associated with the use of HIV VCT, gender, age, and the type of study or department were not associated [7,20]. Additionally, gender was not associated with VCT uptake among Thai university students [5]. Therefore, it is crucial to evaluate this population’s readiness to test for HIV, as well as the factors associated with this willingness, in order to advance our understanding and offer evidence-based suggestions for action. It appears from our survey that HIV VCT utilisation is better in females than males. Female students (71%) denoted the most significant proportion of the population under investigation.

Contrary to what we found, more male students (54.8%) than female students (45.2%) at the Higher Institutions of Learning in Kigali, Rwanda, responded to the HIV VCT study [29]. Additionally, more men than women used the VCT services at Jomo Kenyatta University of Agriculture and Technology [21]. Despite this, more women than men enrol in higher education institutions like universities [30]. Pillay and colleagues contend that females constitute most of the student population and outnumber males at tertiary educational institutions [31]. These could be the cause of the higher percentage of female students (58.8%) in our results who reported having used HIV VCT services in the past. This outcome is similar to a survey conducted among undergraduate students at Ghana’s University of Health and Allied Science Ho campus. In that study, more female students than male students (58.95%) volunteered to test for HIV [8]. Contrary to what we found, more female students (56%) at Thai universities declined to have their HIV status checked than male students [5].

Most of the respondents (52.8%) were between 18 and 21 years old. These results are consistent with the cross-sectional study conducted with university students on determinants of good academic performance, revealing that most students begin their university studies at 18 years old [32]. Therefore, they will likely graduate from undergraduate studies around 21–22 years old, given that most undergraduate programmes at South African universities last between three and four years. Of concern is that a high percentage (52.8%) of these students are not utilising HIV VCT services. Owing to the fact that tertiary students engage in risky sexual behaviours [16] and are prone to sexually transmitted infections such as HIV [17], we need to encourage the 100% usage of HIV services among this age group to achieve an AIDS-free generation. Numerous other studies have also shown that the age of university students has an impact on how often they use HIV VCT, with those between the ages of 18 and 25 using the services more frequently than their peers [11,33,34]. Our results, however, show that while a large percentage of students who answered the questionnaire are between the ages of 18 and 21, the majority of students (63.0%) who are utilising HIV VCT are between the ages of 22 and 25. Our results are consistent with those of Kenyan public Universities, where it was found that the age group between 20 and 24 used the services more frequently [21]. This is nearly identical to a prior survey from Ghanaian undergraduate students, who indicated that most respondents between the ages of 21 and 25 were more likely to use HIV VCT services [35]. Also, literature revealed that this age group is more exposed to HIV education, and hence, they understand the need to know their HIV status [11,35].

Nonetheless, another study found that tertiary students older than twenty-four were three times more likely to pursue HTC than students younger than twenty [22]. The finding that HIV VCT consumption is significantly influenced by age may be explained by the fact that students become more mature and better able to process HIV information. It could also be because young people learn about HIV, and as they get older, they realise how important it is to know their status.

Another significant result of the current survey indicated that nursing students made up the majority of respondents (40.7%). This is not surprising, given the many university nursing students [36]. Due to the frequent demand for nurses in the labour market, job stability, and the overall prestige of the field, nursing has become a more appealing career choice in recent years [37]. In our case, the high number of nursing students might be because the South African government increased the enrolment of the Bachelor of Nursing programme due to the shortage of nurses in the country [38]. Current results revealed that (62.0%) of nursing students are utilising HIV VCT services more than students from other health departments. The majority (95%) of students from nursing training schools in Ghana were utilising HIV services more than non-nursing schools [22]. Relevant to the current finding is that a kind of health department in the university has a substantial impact on HIV VCT consumption. For example, nursing students are more involved in patient activities and health education, consequently increasing their knowledge about HIV prevention and treatment, which enhances their comprehension of the significance of using HIV services.

Furthermore, in the current study, a higher proportion of 4th-level (38.3%) and 3rd-level (30.2%) students used HIV VCT services compared to 1st and 2nd-level students. This finding is similar to a study conducted in Gambia among student nurses and midwives, which discovered that the educational year positively correlates with HIV VCT uptake [39]. Likewise, it was discovered that students in the 4th level are more likely to use HIV VCT than their counterparts [22]. This indicates that the HIV VCT utilisation rate tends to increase with the level of study because people have spent more years learning about HIV.

The survey had some limitations because it did not determine other diverse factors, such as the percentage of students with testing awareness, compared to utilising the VCT services on or off-campus. The results would have assisted with scientific data to enhance the discussion of the current situation.

## 5. Conclusions

In conclusion, it is clear from this study findings that the majority of the university students are aware of the existence of HIV VCT services in the campus health and wellness centre, and it was significantly associated with the utilisation of HIV VCT. However, the university students’ demographic characteristics did not show any associations. Furthermore, they voluntarily utilise HIV services, mainly to know their status. This study recommends that further research on the utilisation of HIV VCT services be conducted in other faculties at the university to build up on the existing knowledge. In addition, another study may evaluate the existing HEAIDS programme to determine its effectiveness. This will assist the stakeholders in developing strategies to help increase the uptake of HIV VCT services among vulnerable University populations. Increasing the utilisation of HIV VCT among University students is crucial in the fight against the HIV/AIDS epidemic in South Africa. It also helps to accomplish the third Sustainable Development Goal, which is to eradicate HIV/AIDS by 2030, among other things. The results of this study have implications for HIV research, policies, and programs.

## Figures and Tables

**Table 1:** Respondents’ demographic characteristics.

Variable	Frequency	Percentage
Gender		
Females	230	71
Males	84	25.9
Others	10	3.1
Age		
≥17	5	1.2
18–21	168	52.8
22–25	152	46
Level of Study		
Level 1	49	15.1
Level 2	53	16.6
Level 3	98	30.2
Level 4	124	38.3
Department		
Nursing	132	40.7
Optometry	57	17.8
Pharmacy	84	25.9
Dietetics	51	15.7

**Table 2:** Respondents utilisation of HIV Voluntary Counselling and Testing.

Variable	Response Rate	Percentage
Awareness of campus health and wellness centre		
No	9	2.8
Yes	315	97.2
Awareness of VCT services offered in the campus health and wellness centre		
No	82	25.3
Yes	242	74.7
Frequency of utilising the HIV VCT services at the campus health and wellness centre		
Every 1 month	42	13.0
Every 3 months	32	9.9
Every 6 months	43	13.3
Every 12 months	65	20.1
More than 3 in 12 months	23	7.1
None	119	36.7
Willingness to utilise the HIV VCT in the campus health and wellness centre		
Agree	195	60.2
Disagree	29	8.9
I do not know	100	30.9
Utilisation of the HIV VCT outside the campus		
No	145	44.8
Yes	140	43.2
No response	39	12.0
If ever voluntarily tested for HIV before		
No	31	9.6
Yes	293	90.4
Reasons for testing		
If engaged in unprotected sex	33	10.2
If getting into a new relationship	31	9.6
If advised by a healthcare provider	27	8.3
If feeling sick	25	7.7
To know your status	208	64.2

**Table 3:** Association between awareness of the Voluntary Counselling and Testing of HIV services offered at the campus health and wellness centre and utilisation of HIV Voluntary Counselling and Testing.

Awareness of the Voluntary Counselling and Testing of HIV Services Offered at the Campus Health and Wellness Centre	Utilisation of HIV Voluntary Counselling and Testing	Chi-Square	*p*-Value
74.7%	65.8%	42.620	<0.001

## Data Availability

Data Availability Statements are available on request.
